# Spatiotemporal Frameworks of Morphogenesis and Cell Lineage Specification in Pre- and Peri-Implantation Mammalian Embryogenesis: Insights and Knowledge Gaps from Mouse Embryo

**DOI:** 10.3390/biology14111596

**Published:** 2025-11-14

**Authors:** Huanhuan Yang

**Affiliations:** School of Medical Sciences, The University of Manchester, Manchester M13 9PL, UK; huanhuan.yang@manchester.ac.uk or xinyuhuanyang@yeah.net

**Keywords:** mammalian embryogenesis, in vitro models, in vivo models, mouse embryo, preimplantation development, peri-implantation development, cell lineage specification, morphogenesis, spatiotemporal dynamics, critical analysis

## Abstract

Research on mammalian embryogenesis has been revolutionised by rapid technological and methodological advancements. This review seeks to synthesise historical and contemporary studies on mouse embryos to provide comprehensive conceptual frameworks of early embryogenesis. Key research findings from live-cell imaging, fluorescence staining, molecular, and sequencing analyses were reassessed, mainly focusing on in vivo and in vitro mouse embryo models. Through critical reassessment, this review first presents historical progress in mammalian models and culture systems for the study of embryogenesis. Subsequently, it elucidates the spatiotemporal progression of morphological events from pre- to peri-implantation across tissue, cellular, and molecular scales. The critical analysis in this review highlights that prevailing and alternative models for pre-implantation cell lineage specification are not mutually exclusive but, rather, describe complementary aspects of embryogenesis. While multiple factors in peri-implantation cell lineage specification are discussed, establishing integrative theoretical models requires further investigation into the crosstalk between different factors during lineage decision-making. Furthermore, this work identifies that precise spatiotemporal dynamics and mechanisms, particularly those governing the initiation, progression, and maintenance of morphological and lineage decisions, remain major knowledge gaps. Lastly, this review provides critical thinking frameworks for assessing current models and defining specific experimental pathways to address enduring unresolved knowledge gaps regarding early embryogenesis.

## Simple Summary

Understanding mammalian embryo development is a fundamental biological pursuit with profound theoretical and translational implications. Advanced in vitro models and cutting-edge methodologies have generated important insights on tissue, cellular, and molecular events regulating pre- and peri-implantation embryogenesis. Simultaneously, divergent data interpretations have led to competitive theories, such as debates over cell lineage specification mechanisms. This environment poses significant challenges for emerging scientists. The need to master field history and complex technologies to pinpoint novel research directions enriching the field is further complicated by a stark reality: challenging prevailing paradigms, even with robust evidence, can lead to loss of support and professional stagnation. Such constraints suppress the open minds and innovative thinking necessary to identify and bridge hidden knowledge gaps in our understanding of early embryogenesis. To overcome these challenges, this review synthesises the historical context, methodologies, and spatiotemporal dynamics of morphogenesis and cell lineage specification in pre- and peri-implantation mouse embryos. This work aims to equip researchers with historical frameworks to effectively understand early embryo development, encourage open-minded critique, illuminate unresolved questions, and provide insights on future research for both experienced and young scientists across interdisciplinary fields.

## 1. Introduction

Mammalian embryo development is a dynamic and intricate process that transforms a single totipotent cell—capable of forming all embryonic and extraembryonic cell types—into a complex multicellular structure. This transformation is orchestrated through pre- and peri-implantation morphogenic events such as compaction, cavitation, and hatching, laying the foundation for early lineage specification and tissue organisation. Understanding these processes holds significance in advancing research on organogenesis, congenital disorders, regenerative medicine, and reproductive health.

This endeavour, initiated in the late 19th century, has relied on the use of tractable mammalian models. Among these models, mice become the cornerstone due to their logistical advantages and the profound conservation of core early developmental principles across mammals. The field has evolved rapidly, driven by sophisticated in vitro culture systems (including stem-cell-based embryo models, which have surged especially since 2019 but are not reviewed here), live-cell imaging, single-cell omics, and advanced molecular tools [1,2,3]. These methodologies have generated unprecedented insights into the multifactorial dynamics of pre- and peri-implantation morphogenesis and cell fate specification, including cell microenvironmental modulation, geometrical constraints, biophysical/biochemical regulation, and cell heterogeneity [4,5].

Despite this accelerated progress, the field has reached a critical point. The proliferation of high-dimensional data from disparate methodological approaches has sparked divergent interpretations and competing theoretical models [6,7,8,9]. A central challenge is the integration of foundational historical knowledge with contemporary discoveries to form a coherent understanding of embryogenesis. Furthermore, innovative thinking and open critique are prevented by a perceived pressure to conform to prevailing models. These challenges are particularly acute for early-career scientists, who must not only master complex technologies and reconcile historical paradigms but also navigate an academic ecosystem that often rewards conformity over consilient research [10].

This review delineates the intricate orchestration of early mouse embryo development. It begins with a summary of historical experimental models and technological advances in the field. Key morphological milestones during pre-implantation development are then charted, followed by an evaluation of both prevailing and alternative models illustrating pre-implantation cell lineage specifications. The discussion then extends to the peri-implantation period, examining its defining morphological events and the dynamic models explaining peri-implantation lineage establishment. By synthesising historical foundations with cutting-edge discoveries, this review aims to construct a coherent framework for quickly grasping early mammalian development while highlighting persistent questions that continue to propel the field forward.

## 2. Mammalian Models for Studying Early Embryogenesis

**Figure 1:**
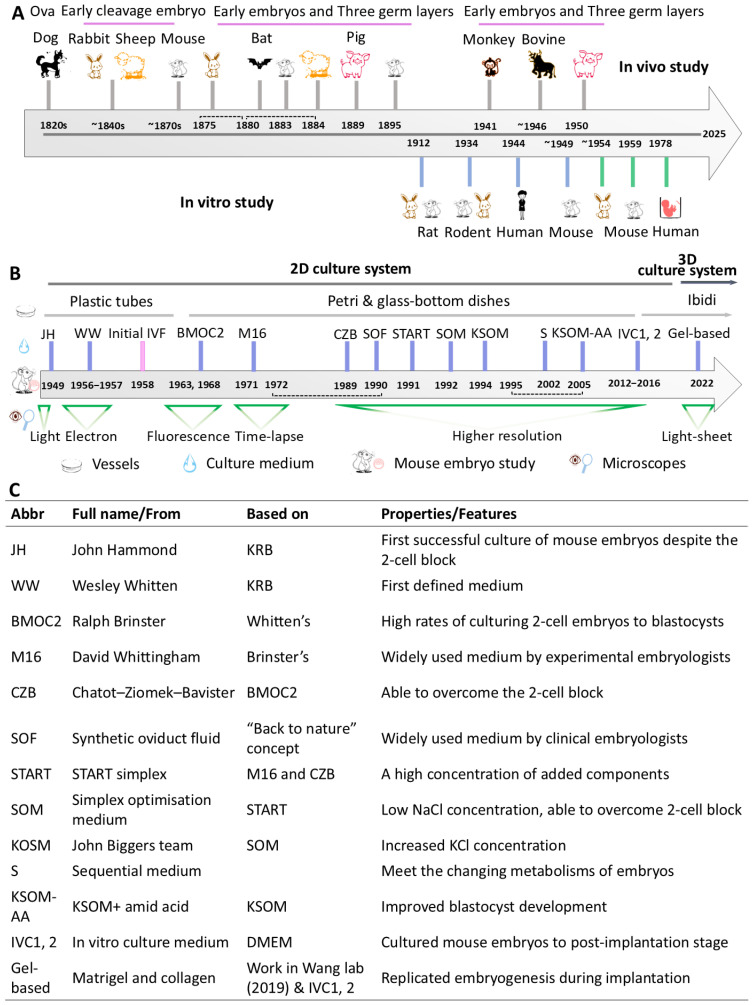
Historical development of mammalian embryo study models and culture systems. (**A**) Timeline of mammalian species used in early embryo studies, both in vivo and in vitro, over the years. “Three germ layers” refer to endoderm, mesoderm, and ectoderm. Species names are shown at the point when they were first studied. Dashed lines indicate the periods of embryo studies for the given mammals. Grey vertical lines show the years of in vivo embryo studies, blue vertical lines represent the years of in vitro culture attempts, and green lines mark the successful in vitro fertilisation (IVF) resulting in live births following embryo transfer. (**B**) Advances in 2D to 3D culture systems (dark grey line), evolution of culture media (blue vertical lines), changes in culture vessel (grey line), and progress in microscopy (green triangle) over time. The pink vertical line indicates the year when IVF mouse embryos successfully resulted in live births after embryo transfer. (**C**) Explanations of abbreviations of media names. KRB means Krebs–Ringer’s bicarbonate solution, in which mouse embryos cannot survive during culture. IVC means in vitro culture. “Work in Wang lab” developed IVC system using matrigel-coated Petri dishes with adapted blastocyst culture medium; see [20,21,30] for details of culture systems.

Unravelling the dynamic processes of pre- and peri-implantation embryo development requires accessible, manageable, and cost-effective animal or embryo models. Over time, numerous mammalian species have been employed to study early embryogenesis. In the late 1820s, the mammalian unfertilised oocyte was first identified in a dog [11]. Nearly half a century later, early embryo development started to be systematically studied in various mammals, including rabbit, bat, sheep, pig, bovines, mice, non-human primate, and human embryos, both in vivo and in vitro (Figure 1A) [12,13,14,15,16,17,18,19,20]. Among these mammals, the mouse—which is focused on in this review—has emerged as a particularly favoured model due to its relatively low cost, short generation times, and easy access to genetically manipulated strains [21]. These studies revealed that while early embryogenesis differs in detail across species, the core principles of self-organisation remain conserved, regulated by intricate cell microenvironment, biophysical/biochemical signalling, and cell plasticity without external blueprint cues [4,5]. Thus, the extensive knowledge gained from the mouse model provides a fundamental framework for exploring and understanding human early development.

Scientific investigation of in vivo pre-implantation mouse embryo development started in the 1870s–1890s [22]. However, optical obstruction limited most in vivo studies to post-implantation stages. In vitro embryo culture enables research on pre- and peri-implantation dynamic events, driven by advances in culture media, environmental control, and live-imaging technologies. The first successful in vitro culture of a mouse embryo was achieved in 1949 using a modified KBF medium [23] (Figure 1B,C). Afterwards, in vitro culture media underwent multiple stages of progress, from M16, SOM, to KSOM—the latter remaining widely used today (Figure 1B,C) [24]. Concurrently, culture vessels have evolved from basic test tubes and plastic Petri dishes to glass-bottom dishes, u-slides, and microfluidics dishes, enhancing experimental control (Figure 1B) [25,26]. Culture systems have advanced from simple 2D platforms to 3D matrices, such as Matrigel-coated slides, to better replicate the in vivo microenvironment for embryogenesis (Figure 1B) [27]. Furthermore, cutting-edge molecular visualisation—like fluorescent biosensors and Förster resonance energy transfer interaction assays—enable tailored spatiotemporal observations of specific molecular, cellular, and tissue activities during early embryo development (Figure 1B) [28,29]. These improvements have been facilitated by parallel advances in imaging technologies, including the shifts from 2D and 3D, phase-contrast to fluorescence microscopy, and static snapshots to real-time monitoring [2,29]. These transformative advancements have greatly expanded our ability to precisely observe and manipulate early embryo development as it unfolds.

## 3. Landmark Morphological Events in the Pre-Implantation Embryo

### 3.1. Fertilisation

**Figure 2:**
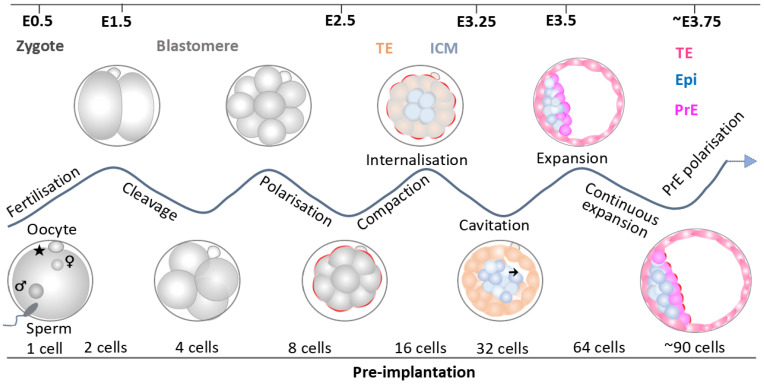
Schematic of mouse preimplantation development. This schematic depicts the process of embryo development from a zygote to a blastocyst. The top and bottom lines show the developmental days in embryonic day (E) and cell numbers, respectively. The waving line labels morphological events. The pentagram in 1 cell marks the second polar body (PB2). Cell lineages are colour-coded: orange for early TE, blue for inner cell mass (ICM) or epiblast (Epi), red for mature trophectoderm (TE), purple/pink for primitive endoderm (PrE).

In mice, following the breakdown of the germinal vesicle (oocyte nucleus in prophase I of meiosis I, MI), the oocyte is arrested at metaphase of meiosis II (MII). Upon stimulation by luteinising hormone, the mature oocyte is released from the ovary into the oviduct, where it can encounter sperm and undergo fertilisation (Figure 2) [31]. During fertilisation, the sperm penetrates the zona pellucida (ZP), an extracellular matrix (ECM) structure surrounding the oocyte, and delivers its condensed paternal chromatin into the oocyte while excluding most of its cytoplasm [31,32]. The oocyte then completes the MII and extrudes the second polar body (PB2) [33]. Both sets of parental chromatins then undergo complex remodelling within the fertilised oocyte, forming maternal and paternal pronuclei and creating a zygote [34,35,36,37]. Afterwards, the pronuclei approach each other without fusion [38].

Fertilisation is not merely the union of gametes, it also sets the stage for subsequent embryogenesis via the transition from meiosis to mitosis. However, the extent to which fertilisation and PB2 influence subsequent developmental processes remains an open question and a subject of ongoing investigation and debate.

### 3.2. Compaction

Following three rounds of cleavage (mitotic division without growth of embryo size as a whole) after fertilisation, the mouse embryo reaches the 8-cell stage and starts compaction (Figure 2). During compaction, blastomeres establish close and flattened cell–cell contact with the disappearance of the visible cell boundaries (Figure 2) [39,40]. This morphological reorganisation transforms the embryo into a compact, mulberry-like structure known as morula (Figure 2) [41]. The primary driving force behind compaction is the changes in the distribution of actomyosin cortex elements, with E-cadherin (E-cad) protein contributing to the maintenance of embryo compaction [42,43]. Furthermore, filopodia and microtubule bridges (MTs) provide structural support and facilitate the transport of adhesion molecule E-cadherin to cell membranes during compaction, respectively, maintaining a flattened cell shape [44,45]. Disruptions of E-cadherin, filopodia, or MT cause distortion of subsequent developmental events and cell lineage specifications [44,45]. However, the full spatiotemporal dynamics of cellular and molecular interactions during compaction remain incompletely understood. Furthermore, the regulation of the compaction rate and its precise relationship with blastocyst formation are yet to be fully elucidated.

### 3.3. Polarisation

At the 8-cell stage, blastomeres begin structural and functional asymmetry; i.e., polarisation, forming apical (cell contact-free) and basolateral (cell contact-facing) domains featured by differential proteins [46,47]. Here, I synthesise existing studies to conceptualise polarisation as a cascade of key molecular events, initiated by phospholipase C (PLC)-mediated hydrolysis of phosphatidylinositol 4, 5-bisphosphate (PIP2) which activates protein kinase C (PKC) [48]. Subsequently, PLC/PKC signalling orients actomyosin near to apical domain via Rho (Ras homolog family) GTPases [48]. Rho cycles between its GTP- and GDP-bound forms to facilitate the localisation of Ezrin and aPKC to the apical domain and direct scribble (Scrib) and lethal giant larvae (Lgl) to the basal region through the aPKC–Partitioning defective protein 6 (Par6)–Par3 pathway [49,50,51]. PKC further promotes the phosphorylation of Ezrin via the mitogen-activated protein kinase/extracellular signal-regulated kinase (Mek/Erk) pathway. Phosphorylated Ezrin binds actin filaments and stabilises within the apical domain during compaction [52]. Concurrently, Rho-associated kinase (ROCK), activated via interacting with Rho, phosphorylates numerous proteins such as myosin light chain, leading to the recruitment of Par3/Par6 complexes to the apical cortex [48,50]. The accumulation of Par3/Par6/aPKC in the apical cortex expels the actin meshwork away from the apical domain, resulting in the formation of a mature apical cap and apicolateral tight junctions [4,47]. These polarised asymmetries within blastomeres not only minimise surface energy and stabilise embryo architecture [53], but also play a critical role in driving later morphological processes and cell lineage specification [43,54].

### 3.4. Cell Internalisation

During cell divisions following compaction and polarisation, some blastomeres localise inside embryos—i.e., cell internalisation—and form the inner cell mass (ICM), while others remain outside and develop into trophectoderm (TE) [43,54,55]. Such cell internalisation persists beyond the 32-cell stage [8,56,57]. Blastomere internalisation is thought to occur through two mechanisms: some cells move inward as a result of cleavage of their progenitors, while others are prompted to relocate due to positional shifts among neighbouring cells, rather than their own cleavage events [57,58,59,60]. Regardless of the possible mechanisms, blastomere internalisation is driven by cell polarity and actomyosin-mediated cortical contraction, with cells exhibiting higher cortical tension and relatively small or no polarity being more likely to internalise [43,54,61,62]. Disruption of actomyosin-mediated cortex tension leads to impaired internalisation [54].

Cell internalisation is thought to be the first significant change in cell position and movement within the embryo that relates to cell lineage specification. However, whether earlier positional and temporal cues could also contribute to cell fate decisions still warrants further investigation. Furthermore, the spatiotemporal dynamics of cell internalisation and its relationships with later embryo development remain incompletely examined.

### 3.5. Cavitation

Approximately around the 32-cell stage, mouse embryos transition from a compact cell mass to a more organised differentiated form known as a blastocyst. During this process, a fluid-filled cavity develops on one side within the embryo while inside cells—i.e., ICM—remain on the other side, both surrounded by TE cells (Figure 2) [63,64]. TE cells exhibit specialised features during blastocyst stages, concentrating proteins like Na^+^/K^+^ ATPases, aquaporins (AQP3, 8, 11), mitochondria, and lipid droplets in the areas where they contact neighbouring cells [39,65]. The spatial adaptations of AQPs, mitochondria, and lipid droplets provide channels for water transport and probably ensure efficient energy metabolism for promoting fluid secretion of TE cells, leading to the initiation of cavitation and blastocyst formation [66,67]. Throughout blastocyst development, embryos exhibit a strong paracellular seal facilitated by the presence of gaps and tight junctions, as well as actin rings between the TE cells [68,69]. Such watertight sealing in the TE epithelium regulates the transport of ions, water, and specific molecules across the cell membrane, mediated by cyclic adenosine monophosphate (cAMP) [70,71]. The cAMP-dependent transport facilitates liquid retention and cavity expansion [70,71]. In addition, fibroblast growth factor 4 (Fgf4) and fluid pressure in the cavity influence TE cell divisions and facilitate PrE cell establishment in vitro [72,73].

Despite recent advances, the spatiotemporal mechanisms involved in initiating, expanding, and regulating embryo cavitation are not fully understood. Furthermore, the influence of the cavity contents and their temporal dynamics on subsequent development remain inadequately explored.

## 4. Key Lineage Specification in the Pre-Implantation Embryo

In the pre-implantation stage, embryonic cells are thought to undergo two major waves of population specification and segregation, resulting in three distinct lineages: TE, the outer cell layer that will form the embryonic part of the placenta; epiblast (Epi), which resides inside the embryo and gives rise to the embryo proper; primitive endoderm (PrE), adjacent to Epi inside the embryo, which develops into visceral and parietal endoderm (VE and PE) and primarily forms the yolk sac (Figure 2) [5,74]. The segregation of TE and ICM at the 16- to 32-cell stage and the differentiation of ICM into PrE and Epi is often termed the first and second cell lineage specification, respectively [75]. The TE cell fate is usually identified by the presence of caudal-type homeobox transcription factor 2 (Cdx2) [76,77,78]. PrE is typically marked with Gata6, Gata4, Pdgfrα (platelet-derived growth factor α), and SRY (sex determining region Y)-box17 (Sox17) [79,80]. Epi fate is associated with octamer-binding transcription factor 4 (Oct4), Nanog, and SRY (sex determining region Y)-box2 (Sox2) [79,81]. It is worth noting that Sox2 is not only Epi/ICM-specific but also marks early TE-derived trophoblast, and Sox2 mutant embryos die of TE defects rather than embryonic defects [82].

### 4.1. Prevailing Models of the First and Second Cell Lineage Specification

#### 4.1.1. The First Cell Fate Decision

**Figure 3:**
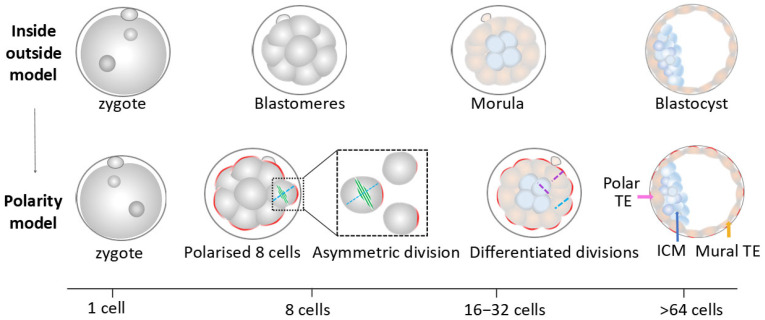
Schematic of two models for the first cell lineage specification. Top inside-outside model represents that cell fate is induced by cell inside/outside positions during the morula stage. Blue stands for inside cells and light pink is coded for outside cells. Bottom polarity model illustrates how inside/outside positions influence cell fates via cell polarity. The green spindle represents the division pattern in the 8-cell stage. Red cell surface refers to polarity after asymmetric cell divisions. Cells with relatively more polarity stay outside while those with less polarity are internalised. Purple is coded for symmetric division in outside and inside cells, and the green dotted line symbolises the asymmetric division in outside cells at the 16-cell stage. Blue represents ICM (inside cells). Light pink/orange represents TE (outside cells), with polar TE (indicated by pink arrow) overlying the ICM and mural TE (indicated by orange arrow) surrounding the cavity. Bottom line shows the developmental timing indicated by cell numbers.

A ‘positional’ model is one of key theories proposed to explain TE and ICM separation. Compaction at the 8-cell stage allows for the generation of inside and outside cells (ICM and TE), traditionally considered the first spatial differences between blastomeres (Figure 3) [83]. These differences in the physical positions of blastomeres are central to the “inside-outside model” for the first cell fate decision, emphasising that embryonic cells possess the ability to sense their positions within embryos and ultimately determine their cell fate based on their location (Figure 3) [84]. Specifically, cells occupying outside positions differentiate into the TE, while cells positioned within the interior of embryos develop into the ICM; this was experimentally tested and confirmed [85,86].

The “inside-outside model” has been extended by the “polarity model”, which highlights the influences of cell polarity on cell fates (Figure 3) [87,88]. In the “polarity model”, daughter cells inherit polarised domains differently from their mother cells during the transitions from 8 to 16 cells and 16 to 32 cells. The cells with an established apical domain are more likely to differentiate into TE, while cells lacking an apical domain or possessing a small apical domain are inclined to develop into ICM (Figure 3) [87,88,89]. This polarity-induced inside-outside cell separation is regulated by actomyosin activity [43,54], as discussed above. Specifically, actomyosin contractility generates high cortical tensions, driving embryonic cells to move inward as ICM, while cells with low cortical tensions contribute to TE. The “polarity model” has been continuously updated to integrate multiple factors, including cell position, cell polarity, cell division pattern, and mechanical force with the Hippo pathway [46,90,91,92]. However, the detailed mechanisms linking these factors still require further investigation.

#### 4.1.2. The Second Cell Lineage Specification

##### Main Models for the Second Cell Fate Decision

Several models have emerged to explain the second cell fate specification. The first, known as the “positional model”, suggests that the final differentiation of ICM into either Epi or PrE is determined by cell position [93]. Specifically, ICM cells positioned along the cavity differentiate into the PrE cell lineage, whilst the deeper ICM cells undergo specification towards the Epi cell fate [93].

**Figure 4:**
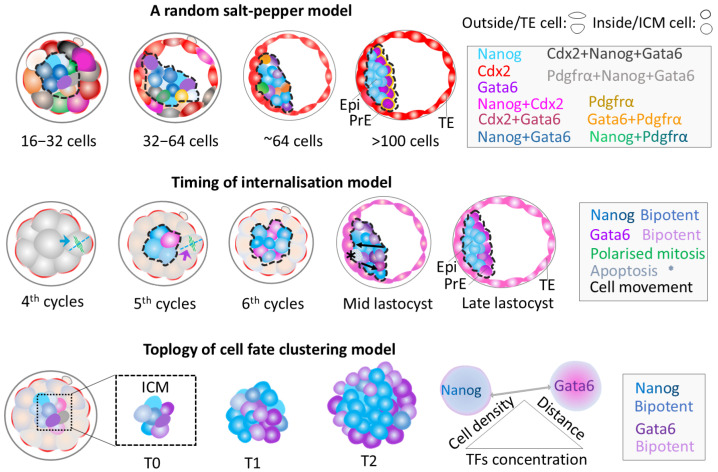
Three main models explaining the second cell lineage specification. The top panel shows a three-step model. Cells drawn in a relatively round shape within dashed black circle represent the inside or ICM cells, while those drawn in spindle- or fan-like shapes outside the dashed black circle represent the outside or TE cells. Different transcriptional factors (TFs) and their co-expressions are colour-coded as shown in the figure and the accompanying box. Different shades of a colour represent TFs expression levels, with darker hues indicating higher expression and lighter indicating lower expression. The middle panel exhibits the position induction model (i.e., timing internalisation models). Cells within the black dashed line stand for inside or ICM cells, while those outside the line represent the outside or TE cells. The green spindle means division pattern during morula stages, and blue and purple arrows in the fourth and fifth division rounds highlight daughter cells that preferentially produce more Epi (Nanog-positive, blue) and PrE cells (Gata6-positive, purple) after the asymmetric division. Asterisks in the embryo and the box refer to cell apoptosis. Black curve arrow stands for cell movement direction. The bottom panel represents the dynamics of the topology of local cell fate clustering and the relations between cell clustering and three factors: cell neighbouring density, cell distance and TFs expression concentration. T0, T1 and T2 respectively represent 24 h, 48 h, 72 h of culture time. Different cell fate clusters of Epi- and PrE-like cells are represented by dark and light blue (Nanog) as well as dark and light purple (Gata6), respectively.

Different from the one-step positional model mentioned above, a “three-phase model” describes a multi-step process for ICM differentiation (Figure 4) [94]. In the first step, during the 16-cell to 32-cell stage, different cell lineage-related transcriptional factors (TFs) exhibit overlapping expression patterns within embryonic cells [94]. In the second step, lineage-specific transcription factors are expressed exclusively, resulting in a random salt-and-pepper arrangement of Epi- and PrE-precursor cells between the 32- to 64-cell stage [94,95]. The third step involves dynamic cell movements, leading to the final sorting and spatial segregation of Epi/PrE cell lineage around the 128-cell stage [94,96]. In this model, it is unknown how initially co-expressed TFs in the first step are subsequently restricted to specific cell types in the second step, or how the “random salt-pepper” pattern of Epi/PrE precursors is gradually organised into distinct Epi and PrE layers in the third step.

Contrasting with the “random salt-pepper” sorting pattern of Epi- and PrE-precursors seen in the multi-step model, which is inferred from discontinuous single timeframes, a “timing of internalisation” model is built on the continuous cell track. This timing of internalisation model proposes that internalised cells from the fourth round of division are more likely to settle deep in the ICM, favouring Epi development while internalised cells from the fifth-round division tend to be near the ICM surface, promoting the PrE lineage (Figure 4) [8,97]. This internalisation-timing-associated cell sorting was previously thought to be the result of inheritance of higher levels of fibroblast growth factor receptor 2 (Fgfr2) from surrounding external cells [8]. However, a recent study has demonstrated that Fgfr1, rather than Fgfr2, is essential for ICM lineage specification and PrE formation [98]. Thus, it remains unclear how cells from the fourth and the fifth round of divisions (occurring at the compacted morula stage) tend to respectively develop into Epi and PrE cell layers (fully sorted during the late blastocyst stage).

Additionally, I framed recent findings as a “topology of cell fate clustering” model. This model redefines cell lineage specification by considering geometrical relationships and composition of 3D cellular neighbouring communities (Figure 4) [99,100,101]. Supporting this, studies in embryos and ICM organoids exhibit that Epi/PrE precursor positioning is mainly influenced by proximity to other precursors, and cell packing density depends on the levels of Gata6 and Nanog expression [100,101]. Specifically, outer-layer cells in ICM of embryos and organoids show high levels of the PrE-precursor marker Gata6, and inner-core cells express the Epi-precursor marker Nanog, with few in the organoid core expressing Gata4 (Figure 4) [99,101,102]. Furthermore, the number of neighbouring PrE precursors depends on the levels of Gata6 within adjacent cells, whereas Epi precursors consistently have nine neighbours during the 32- and 64-cell stages [101,102]. This process may involve intercellular inhibition and homogenous genetic activation, which collectively reinforces the maintenance of similar cell fates across cell populations (Figure 4). However, the detailed spatiotemporal mechanisms by which the expression levels of specific TFs, their concentration gradients, neighbouring cell numbers, and their distances regulate cell fate decisions and sorting remain incompletely understood.

While each of the aforementioned models offers valuable insights into the intricate processes of ICM differentiation into Epi and PrE, they are also limited in capturing the full dynamics of cell fate determination. Table 1 provides a comparison of the similarities and differences among the models discussed above. Specifically, the “salt-pepper” model provides a framework for understanding the distribution of Epi- and PrE-related cells at certain stages; however, its reliance on static snapshot data precludes the reconstruction of dynamic developmental trajectories of individual cells, leaving its proposition of a ‘random’ patterning mechanism inadequately supported. The “timing of internalisation” model directly addresses this shortcoming by demonstrating the influence of developmental history of 8- to 32-cell internalisation on Epi and PrE specification, challenging the idea of ‘randomness’ in the ‘salt-pepper pattern’. However, the proposal of primacy to Fgf signalling for Epi/PrE separation in the ‘timing of internalisation” model has been recently refined by research showing a more complex interplay of pathways. The ‘topology of cell fate clustering’ model provides insights into the importance of positional neighbouring cell interactions in cell fate decisions, yet is constrained by static data.

##### Synthesised Theoretical Model for the Second Cell Fate Decision

Based on the critical analysis of three main models for the second cell lineage specification, a synthesised theoretical framework integrating all discussed models can be proposed here. At the morula stage, cells from different cell division rounds may exhibit varying spatial patterns and gradients of co-expressed TFs in the late morula and blastocyst [94,95,96,97], potentially reflecting the distinct timing of internalisation of cells expressing these factors. Notably, other unknown factors may also contribute to this process, as only two division rounds (the fourth and fifth) are insufficient to fully explain the diversities of co-expression TFs patterns discussed in the salt-pepper pattern.

In early to mid blastocysts, the co-expressed TFs in embryonic cells undergo up- or down-regulation by multiple factors. These may include the co-expressed TFs themselves, historically stored players from early stages and de novo synthesised responsive factors (such as Fgfs and their receptors) in cells, extracellular matrix, or blastocoel cavity [29,73,98]. Within the embryonic cell community, these factors may regulate co-expressed TFs through negative or positive feedback loops within the same factors, synergistic or antagonistic interactions between different factors, or via autocrine, paracrine, and telecrine signalling. Consequently, co-expressed TFs become restricted to Epi- or PrE-like cells, which exhibit a sectional salt-pepper pattern in the spatial distribution of Gata6 and Nanog expressions at single time points [94,95,96]. From a longitudinal spatiotemporal perspective, Nanog-positive Epi-like and Gata6-positive PrE-like cells present specific trackable origin-differentiation trajectories [8,97].

Throughout the blastocyst, regulated by the feedback loops, responses, and signalling from multiple factors, Epi-like and PrE-like or bipotent cells possess distinct behaviour and functional potentials. These cells may undergo directional/passive movement and programmed cell death within the ICM cell community [8,94,97,99,100,101,102], potentially leading to the complete separation of Epi/PrE lineage layers and tissue packing.

This integration theoretical framework requires experimental confirmation by investigating different spatiotemporal factors/signalling, as well as their connections and underlying mechanisms.

### 4.2. Alternative Interpretations of the First and Second Cell Lineage Specification

In contrast with the above discussed models specifying the first and second cell lineage specification during the 8- to 16-cell stages and around the 32-cell stage, respectively, alternative theories and studies propose that embryonic patterning is initiated earlier. The “pre-patterning model”, proposed by Dalcq [103], hypothesises that non-uniformly distributed maternal determinants within the zygote could be segregated asymmetrically into daughter cells to specify cell fates of TE and ICM [103]. Subsequently, models describing that polarity-like features bias cell fate decisions of TE/ICM and that time and positions of 8- to 32-cell asymmetric divisions induce cell fate decision of PrE/Epi have been suggested [6,97]. Although such early-patterning models have been historically debated, challenged, or largely ruled out, increasing evidence suggests unequal blastomere features associated with cell fates emerge as early as the 2- and 4-cell stages [104,105,106,107,108]. Therefore, these aforementioned potential models essentially need re-evaluation. Additionally, the links between the events of early embryonic cells before morula and the first and second cell lineage specifications remain incompletely understood. Moreover, during pre-implantation, TE cells were categorised into polar TE (covering the ICM cells) and mural TE (surrounding the cavity). The latter are thought to arise from the flow of polar TE cells [109,110]. This categorisation is based on the spatial relation between TE cells and the cavity. However, whether this TE categorisation and lineage expansion are the only explanation remains poorly studied. The spatiotemporal nature, properties, and interplay between the two TE cell groups are not yet fully explored.

## 5. Key Morphological Changes in Peri-Implantation Mouse Embryo

### 5.1. Embryo Hatching

**Figure 5:**
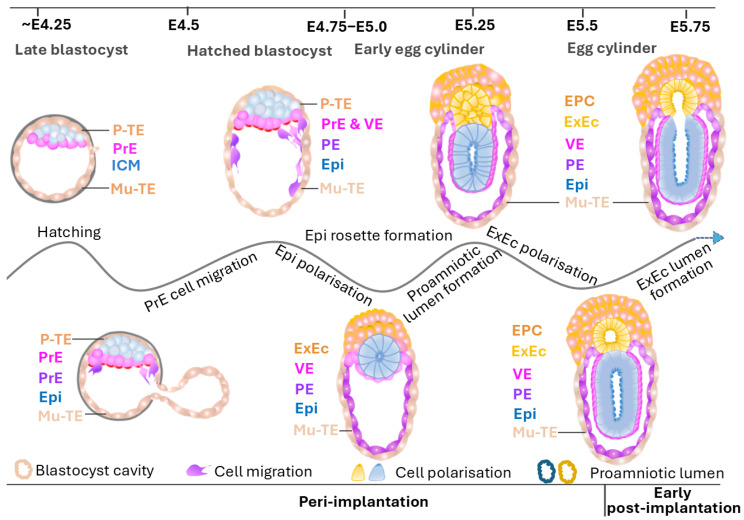
Schematic of morphological changes in peri-implantation embryos. This schematic depicts the process of embryo development from hatching to egg cylinder formation during the peri-implantation stage. The top line shows the developmental days in embryonic day (E). The waving line labels the morphological event roadmap. Cell lineages are colour-coded: dark orange for ectoplacental cone (EPC), orange for extraembryonic ectoderm (ExEc), blue for ICM or epiblast (Epi), wood colour for early TE—polar TE (P-TE) and mural TE (Mu-TE) or late Mu-TE, light purple/pink for PrE/visceral endoderm (VE), purple for parietal endoderm (PE), blue for Epi.

At the late blastocyst stage, embryos attain readiness to break free from the surrounding ZP, a process termed embryo hatching (Figure 5) [111]. In vitro, hatching is facilitated by ZP-lytic enzymes (e.g., strypsin and luminal serine proteinase), which lead to a localised ZP hole for blastocyst escape; in particular, these hatching enzymes diminish the thickness of the whole ZP in vivo [112,113]. The cells initiating this escape can be both polar and mural TE; however, hatching occurring adjacent to mural TE relates to higher implantation and live birth rates, probably driven by transcription factor 24 (Tcf24)- and distal-less homeobox 3 (Dlx3)-regulated site-specific expression of immune genes such as prostaglandin-endoperoxide synthase 1 (*Ptgs1*), lysozyme 2 (*Lyz2*), interleukin 1 alpha (*Il-α*), complement factor B (*Cfb*), and Cd36 molecule-fatty acid translocase (*Cd36*) [114,115,116,117]. Additionally, the expansion of the fluid-filled cavity thins the ZP, facilitating its rupture [118]. Culture media with reduced essential amino acids and increased non-essential amino acids also enhances hatching by accelerating blastocyst formation [119,120]. Moreover, extracellular vesicles and cytokines such as interleukin 1 beta (IL-1β) and interleukin 6 (IL-6) generated by blastocysts promote hatching, with the vesicles regulating potential embryotrophic factors [121,122,123]. Nevertheless, the detailed dynamics and mechanisms of hatching, as well as its influence on subsequent embryogenesis, remain incompletely understood, particularly in vivo.

### 5.2. Cell Migration

Following the late blastocyst stage, PrE- and TE-derived cells undergo active migration, a process of cell movement with remarkable changes in cell morphology and molecular characteristics (Figure 5) [124,125,126]. Through synthesis of existing research, I observed that in late blastocysts, certain PrE-derived cells extend protrusions and migrate towards the mural TE, where they scatter and line the mural TE before developing into parietal endoderm (PE) (Figure 5) [110,124,127]. The remaining PrE cells in contact with Epi—i.e., visceral endoderm (VE)—move to cover expanded polar TE cells [110,127,128]. Initially, PE cell migration was speculated to occur through transdifferentiation from VE via the marginal zone endoderm (MZE), a transient layer lining the TE and PrE (further discussed below) [129]. However, whether this proposed VE-transdifferentiated PE migration pathway is accurate, and how it differs from PrE-derived PE-like/PE migration, requires further investigation. During implantation, differentiated migratory trophoblast cells—i.e., trophoblast giant cells (TGCs)—migrate and invade into the uterus wall via filopodia and lamellipodia extended at their leading edges [130,131,132,133]. Trophoblast migration is regulated by keratin 18 (Krt18), which stabilises cell surface E-cadherin, and by forkhead box M1 (FoxM1), which promotes the expression of migration-related genes such as pyruvate kinase L/R (*Pkl4*), vascular endothelial growth factor (*Vegf)*, and matrix metallopeptidase 2 (*Mmp2*) [134,135]. Given the phenotypic similarity of migrating TE cells to endothelial–mesenchymal transition (EMT), their migration during peri-implantation are proposed to involve EMT-like mechanisms [136]. However, again, the detailed spatiotemporal dynamics and mechanisms of PrE-derived and TE-derived cell migration throughout implantation await further investigation.

### 5.3. Egg Cylinder Formation

Since peri-implantation, embryos remarkably transform from a spherical blastocyst to an elongated egg cylinder structure (Figure 5) [137]. Specifically, following attachment to the uterus in a manner regulated by differentiated mural TE cells in mice, hatched blastocyst gradually flattens with the cavity collapsing [124,138,139]. Simultaneously, single-layer polar TE cells undergo morphological remodelling and fast proliferation. This generates a pushing force towards Epi cells, facilitating their rapid growth and rearrangement into a rosette-like structure located above a corresponding rosette of central polar trophoblast; i.e., extraembryonic ectoderm (ExEc) [111,122,124,140]. In contrast, peripheral trophoblast cells gradually form the ectoplacental cone (EPC). Both the rosette-like epiblast and ExEc are covered by VE cells, defining two regions of the egg cylinder: a distal region, featuring epiblast and VE, and a proximal region, characterised by central trophoblast (Figure 5) [122,141]. The late stage of egg cylinder formation involves proamniotic cavity formation and expansion, which is discussed below.

### 5.4. Proamniotic Cavity Formation

During the establishment of Epi and trophoblast rosettes, Epi and polar TE cells undergo polarisation marked by the apical localisation of proteins Par, aPKC, myosin II, F-actin, catenin, and E-cadherin (Figure 5) [26,122]. This polarisation initiates proamniotic cavity formation within both rosettes via regulation of anti-adhesive molecules such as CD43 family and re-organisation of E-cadherin [122]. This molecular reorganisation leads to low intercellular adhesion sites at apical domain of cells for ion and water transport to form small lumens, probably directed by bone morphogenetic protein (Bmp3), transforming growth factor-beta (Tgf-β), Sma and Mad related protein (Smad) pathways, β1-integrin signalling, and podocalyxin exocytosis [26,122,140,142]. The initiated lumens then expand via paracellular fluid flow during the surrounding Epi cell mitosis [122]. The fluid for this expansion is sourced from the blastocoel [122]. Subsequently, expanded cavities in the centre of both polarised TE and Epi mass gradually fuse to form a whole proamniotic cavity (Figure 5) [122,140]. However, due to ‘black box’ (embryo implantation)-associated technical challenges, the in vivo dynamics of proamniotic changes and mechanisms are still understudied.

## 6. Key Cell Lineage Establishment in Peri-Implantation Mouse Embryo

### 6.1. PrE Differentiation into Extraembryonic Endoderm

**Figure 6:**
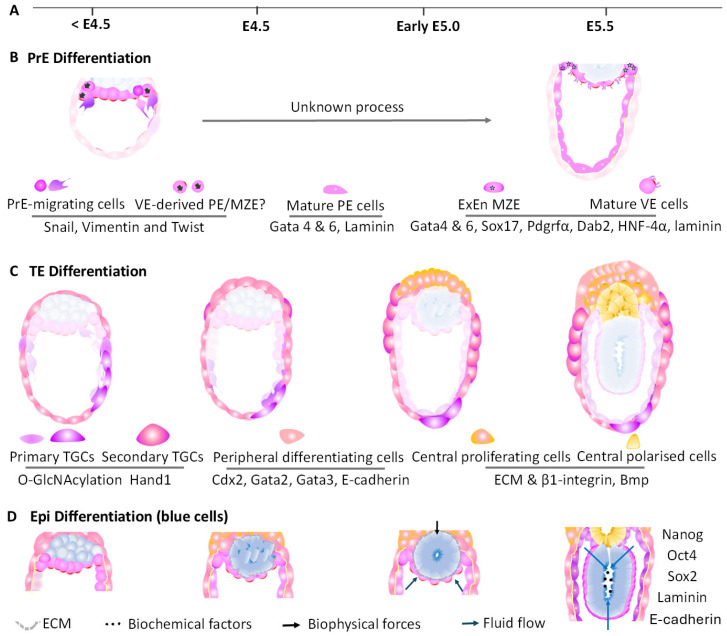
Schematic of mechanisms directing cell lineage specification in peri-implantation embryos. (**A**) Timelines of embryo developmental stages from embryonic day (E). (**B**) PrE cell differentiation with molecular regulators. Different subsets of PrE cells with specific morphologies and TFs are colour-coded as shown in the figure. Cells drawn with black arrows or asterisks represent the potential MZE located between VE, PE, polar TE, and Epi. The long grey arrow indicates the process that is not yet fully elucidated. (**C**) illustrates TE cell differentiation. Distinct subsets of TE cells are denoted by different colours and shapes as shown in the figure. (**D**) depicts Epi (blue cells) differentiation under regulation of ECM, biochemical, and biophysical factors. The yellow curve line between outside TE-derived cells and inside PE cells indicate basement membrane.

After peri-implantation, PrE differentiates into PE and VE, constituting extraembryonic endoderm (ExEn) [124,127,143]. The aforementioned MZE was initially suggested as a distinct pre-implantation endoderm subset, located at the boundary region of VE, PE, polar TE, and Epi, covering the EPC (Figure 6A,B) [129,144]. Previously proposed as an entry point of the VE-PE transition [129,144], the MZE exhibits both VE and PE morphologies, with gradient expressions of Sox17/SRY (sex determining region Y)-box7 (Sox7)/Gata6 from the VE to PE region [144]. However, again, whether the MZE truly represents a transient population transdifferentiated from VE into migrating PE needs re-evaluation. PE formation, marked by high Gata6, is regulated by Sox17 during initial stages to prevent premature cell migration [80,137,144]. Other factors associated with PrE migration and PE differentiation may include Snail, parathyroid hormone related peptide (PTHrP), and Wnt (such as Wnt 6, 7) [145]. Microvilli-characterised VE differentiation, morphology, and maturation are modulated by disabled homolog 2 (Dab2), Gata6, Gata4, Sox7, Sox17, Fgf, and Bmp signalling [145,146,147]. Recently, contents in the cavity, such as Fgf4, have been found to facilitate VE differentiation [73]. Left-right determination factor 1 (*Lefty1*), driving future VE subset (distal VE) along with *Cer1*, may facilitate VE differentiation in the pre-implantation ICM [74]. Despite these insights, the detailed interactions of these factors and mechanisms governing the initiation, progression, maturation, and maintenance of both PE and VE lineages require further study.

### 6.2. TE Cell Differentiation into Extraembryonic Ectoderm

During implantation, the cessation of cell flow from polar to mural TE allows for formation of the tissue boundary between these two regions, a process modulated by actomyosin and Fgf-Fgfr1 signalling [110,148]. With this boundary, proliferating polar TE cells differentiate into distal ExEc, marked by *Eomesodermin* (*Eomes*), *Sox2* and *Fgfr2*, and proximal EPC, featuring *Ascl2* expression and downregulation of *Eomes* and *Sox2* (Figure 6A,C) [110,140,149,150,151]. The E26 transformation-specific family of transcription factors (Ets) and E74-like Ets transcription factor 5 (Elf5) initially enhance ExEc proliferation and then drive proximal EPC and the distal ExEc axis, corresponding to increasing sensitivity to their activities [152]. This axis is refined by β1, β3, and β5 integrins, which transmit polarising cues from the basement membrane between PE and TE to specify distal ExEc. Distal ExEc-derived Bmp4 promotes EPC commitment [151]. EPC formation also requires the regulation of Kat8 (lysine acetyltransferase 8)-mediated H4K16 acetylation, which upregulates *Cdx2* expression to maintain the stemness of TE during implantation (i.e., trophoblast) and potentially promotes TE differentiation into EPC in turn [153]. Furthermore, the closure of the EPC cavity (note that the detailed mechanism of this cavity’s formation remains unclear) requires the regulation of ExEc-derived Efr, which is produced upon Fgf/Erk attenuation and in turn reproduces Fgf [154]. In contrast, mural TE cells transform into the *Eomes- and Elf5-*expressing trophoblast cell layer, with heart and neural crest derivatives expressed 1 (*Hand1*)-expressing primary TGCs exhibiting great invasive activities during implantation (note that secondary TGCs stem from EPC) (Figure 6A,C) [142,155,156]. The EMT-associated transition of TE to an invasive phenotype of TGCs is driven by O-GlcNAcylation, cadherin-mediated pathway, genes of retinoblastoma (*Rb*), *and Eomes*, and is not solely dependent on *Cdx2* (Figure 6A,C) [138,139,157,158,159,160,161]. Although recent studies have continued to identify new genes involved in this process [156], the precise spatiotemporal dynamics and mechanisms that initiate, control, and terminate differentiation of peri-implantation TE require future investigation.

### 6.3. Epi Development into Rosette-like Epi

Fast proliferation of polar TE cells during implantation generates physical forces to drive the Epi cell morphology into a cup-like morphology (Figure 6A,D) [162]. Concomitantly, the laminin exerted from PrE directs the polarisation of Epi cells which are still characterised by Oct4, Sox2, and Nanog during peri-implantation [149,163]. Contents of the proamniotic cavity, such as furin and Nodal, as well as potential factors such as Fgf2 and Bmp4, contribute to Epi proliferation and rosette formation [122,164,165]. Other pathways participating in the maturation of functional Epi include Tgfβ, glycogen synthase kinase (Gsk3), and E-cadherin signalling [146,149,166]. However, the detailed molecular interactive web among different players in peri-implantation Epi differentiation over time remains unclear.

## 7. Conclusions

**Figure 7:**
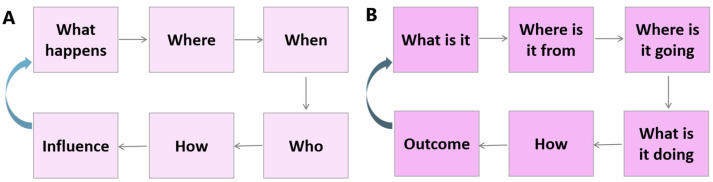
Schematic of two 5W1H frameworks for critical analysis. (**A**) illustrates a framework for analysing events or phenomena (e.g., in embryogenesis or experimental findings), prioritising the identification of core elements (e.g., key players) and their dynamic interactions. It progresses through sequential inquiry into “what happens,” “where,” “when,” “who (which cell or which molecule),” “how,” and what “influence,” forming an iterative cycle that integrates spatial and temporal dimensions of the observed subject. (**B**) focuses on a specific entity (e.g., a cell, a molecular player, or cellular component), tracing its origin (“where is it from”), trajectory (“where is it going”), functional role (“what is it doing”), and resulting impact (“outcome”). This framework emphasises the entity’s dynamic changes within its environment across space and time and the underlying mechanisms or pathways. Curved blue arrows in both panels indicate that each framework operates in a spiral iterative manner, supporting progressive and critical analysis across different scales.

This review endeavoured to chart the developmental trajectory of mammalian embryogenesis, tracing the journey from a zygote to a pre-gastrulation embryo by synthesising foundational knowledge with contemporary advances. From this synthesis, a central theme has come into focus: the intricate interplay between cellular position, polarity, molecular factors, mechanical factors, developmental histories, and temporal dynamics is critical for driving fate decisions. The recognition of this interplay, achieved through technological innovation, has been instrumental in refining the prevailing current models. Despite these advances, the detailed spatiotemporal process and mechanisms of early embryogenesis remain fragmented and poorly understood. Achieving a comprehensive theory requires a critical, open-minded, and systematic approach that addresses fundamental questions—*what happens, when, where, who, how, and influence;* and *what is it, where is it from, where is it going, what is it doing, how, and outcome*—in an iterative spiral manner across different biological scales (Figure 7). I exemplify this framework by applying it to pivotal processes like cavitation and PrE development. For instance, a complete understanding of cavitation requires moving from description to the precise molecular events (what), their exact timing (when), the spatial initiation and expansion of the cavity (where), the specific responsible cells and factors (who), the underlying biophysical mechanisms (how), and the functional imperative/roles (influence). Similarly, investigating the PrE requires determining its compositional heterogeneity (what), tracing its lineage origins and specification signals (who/where from), mapping its positional dynamics and migration (where is it going), understanding how its secretory and metabolic functions maintain adjacent tissues (how), and establishing the developmental consequences of its activity (outcome). Through such rigorous and structured inquiry, this review aims not only to consolidate current knowledge but also to provide a conceptual scaffold that guides future research toward an integrated model of embryogenesis.

## 8. Future Directions

A paramount unresolved question is whether the current analytical techniques are sufficiently comprehensive to detect the complete suite of biomolecular, biophysical, biomechanical, and bio-geometrical cues that govern blastomere fate throughout pre-implantation. Until such a complete spatiotemporal profile is achieved, experimental manipulations risk producing artefacts and/or leading to fragmental understanding of embryogenesis through altering unmeasured variables, thereby confounding the very mechanisms we seek to understand. Therefore, future progress necessitates concerted interdisciplinary efforts aimed at: (1) elucidating the precise spatiotemporal dynamics of key morphogenetic events; (2) developing integrated models to reconcile lineage specification theories; and (3) decoding the signalling networks orchestrating peri-implantation differentiation. Only by collectively addressing these gaps can the field construct a robust, system-level framework that is continually refined through technological and conceptual advancements.

## Figures and Tables

**Table 1:** Comparative analysis of three main models for the second lineage specification.

	Analysis	Cell Sorting Theory	Stage	Models Used	Main Methods	Unanswered Questions
Models						
Salt-pepper (Three steps)		Random sorting	16-cell to 128-cell	Mouse embryo in vivo in vitro	Sectional imaging	How does each step happen? What happens between steps?
Timing of internalisation		Set by division waves	8-cell to 128-cell	Mouse embryo in vitro	Non-invasive cell tracking	How morula division sets cell fates in blastocyst
Cell fate clustering		Community unity	Morula to blastocyst	ICM organoid Embryo in vivo	Sectional imaging Computation	Interaction mechanisms of cell distance, numbers &TFs

## Data Availability

No new data were created or analysed in this study. Data sharing is not applicable to this article.

## Abbreviations

The following abbreviations are used in this manuscript:

AQP	Aquaporin
Bmp	Bone morphogenetic protein
cAMP	Cyclic adenosine monophosphate
Cdx2	Caudal-type homeobox transcription factor 2
*Cd36*	CD36 molecule (Fatty acid translocase)
*Cfb*	Complement factor B
*Dab2*	Disabled homolog 2
Dlx3	Distal-less homeobox 3
ECM	Extracellular matrix
Elf5	E74-like ETS transcription factor 5
EMT	Epithelial-to-mesenchymal transition
*Eomes*	Eomesodermin
EPC	Ectoplacental cone
Epi	Epiblast
ErK	Extracellular signal-regulated kinase
Ets	E26 transformation-specific family of transcription factors
ExEc	Extraembryonic ectoderm
Fgf	Fibroblast growth factor
Fgfr	Fibroblast growth factor receptor
FoxM1	Forkhead box protein M1
Gsk	Glycogen synthase kinase
H4K16	Histone H4, lysine 16
*Hand1*	Heart and neural crest derivatives expressed 1
ICM	Inner cell mass
*Il-1α*	Interleukin 1 alpha
Il-1β	Interleukin 1 beta
Il-6	Interleukin 6
Krt18	Keratin 18
Lgl	Lethal giant larvae
*Lefty1*	Left-right determination factor 1
*Lyz2*	Lysozyme 2
Mek/Erk	Mitogen-activated protein kinase/extracellular signal-regulated kinase
*Mmp2*	Matrix metallopeptidase 2
MTs	Microtubule bridges
MZE	Marginal zone endoderm
Oct4	Octamer-binding transcription factor 4
Par	Partitioning defective
PB2	Second polar body
Pdgfrα	Phosphatidylinositol 4,5-bisphosphate
*Pkl4*	Pyruvate kinase L/R
PKC	Protein kinase C
PLC	Phospholipase C
PrE	Primitive endoderm
*Ptgs1*	Prostaglandin-endoperoxide synthase 1
PTHrP	Parathyroid hormone-related protein
*Rb*	Rho-associated protein kinase
Scrib	Scribble
Smad	Sma and Mad related protein
Sox2	SRY (sex determining region Y)-box2
Sox7	SRY (sex determining region Y)-box7
Sox17	SRY (sex determining region Y)-box17
Tcf24	Transcription factor 24
TE	Trophectoderm
TGC	Trophoblast giant cell
Tgf-β	Transforming growth factor-beta
*Vegf*	Vascular endothelial growth factor
Wnt	Wingless-related integration site
ZP	Zona pellucida
